# Vacuum Nanohole Array Embedded Phosphorescent Organic Light Emitting Diodes

**DOI:** 10.1038/srep08685

**Published:** 2015-03-03

**Authors:** Sohee Jeon, Jeong-Hwan Lee, Jun-Ho Jeong, Young Seok Song, Chang-Ki Moon, Jang-Joo Kim, Jae Ryoun Youn

**Affiliations:** 1 Research Institute of Advanced Materials (RIAM), Department of Materials Science and Engineering, Seoul National University, Seoul, 151-744, Korea; 2 Department of Nano Manufacturing Technology, Nano-Convergence Mechanical Systems Research Division, Korea Institute of Machinery and Materials, Daejeon, 305-343, Korea; 3 Department of Fiber System Engineering, Dankook University, Yongin, Gyeonggi, 448-701, Korea

## Abstract

Light extraction from organic light-emitting diodes that utilize phosphorescent materials has an internal efficiency of 100% but is limited by an external quantum efficiency (EQE) of 30%. In this study, extremely high-efficiency organic light emitting diodes (OLEDs) with an EQE of greater than 50% and low roll-off were produced by inserting a vacuum nanohole array (VNHA) into phosphorescent OLEDs (PhOLEDs). The resultant extraction enhancement was quantified in terms of EQE by comparing experimentally measured results with those produced from optical modeling analysis, which assumes the near-perfect electric characteristics of the device. A comparison of the experimental data and optical modeling results indicated that the VNHA extracts the entire waveguide loss into the air. The EQE obtained in this study is the highest value obtained to date for bottom-emitting OLEDs.

OLEDs intrinsically provide higher energy efficiency than other light sources, and this efficiency has been increased significantly through the use of phosphorescent emitters and charge transport materials. A nearly 100% charge balance can be realized with proper vertical arrangement of these materials in a device. Recently, Park et al. demonstrated that OLEDs could yield extremely high efficiencies and low roll-off if produced with an exciplex forming co-host in the emitting layer (EML)^1^,^2^. They produced a highly efficient OLED with an EQE of greater than 29% without the use of any additional structures for light extraction. Because their OLEDs had an ultrahigh EQE of 29.1%, the EQE had nearly reached the theoretical limit^3^. To the best of the authors' knowledge, this value is one of the highest EQEs for bottom-emitting green phosphorescent OLEDs; however, 70% of the internal light was still wasted by the substrate, organic layers, and transparent electrode even in the case of the highest EQE of 29.1%, which was produced with an internal efficiency of 100%. Although the internal efficiency reached 100%, the external efficiency of the OLED can be further increased so that it can be applied in common industrial applications, such as general lighting.

It is possible for loss modes to be converted into the air mode by controlling the photonic structures of the interior and exterior of the OLED. Thus far, various methods have been proposed to convert the waveguide and glass modes into the radiation mode (e.g., textured surfaces^4^,^5^, microlens arrays^6^,^7^,^8^, scattering media^9^, Bragg gratings^10^,^11^, and low-index grids^12^). As a result, extraction efficiency has continued to be improved. In general, loss from the glass substrate can be prevented by modifying the glass surface, whereas waveguide loss can be extracted by inserting a photonic structure between the glass and transparent anode.

In this study, we attempted to increase the OLED EQE by adding a photonic structure to the device. With this approach, extremely high-efficiency OLEDs with an EQE of over 50% and low roll-off were achieved by inserting vacuum nanohole arrays (VNHAs) into phosphorescent OLEDs (PhOLEDs). The extraction enhancement was then quantified in terms of EQE by comparing the experimentally measured results with those of an optical modeling analysis, which assumed the near-perfect electric characteristics of the device.

## OLED Efficiency

In general, the EQE of an OLED is expressed by the following equation^13^,^14^,^15^:
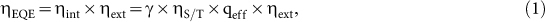
where γ is the charge balance factor, η_S/T_ is the singlet-triplet factor (η_S/T_ = 0.25 for fluorescent, η_S/T_ = 1 for phosphorescent emitters), q_eff_ is the effective PL quantum yield, and η_ext_ is the extraction efficiency of the emitted light. OLED efficiency is determined by complex physics because all terms of the equation affect each other^16^,^17^. If the value of *η_int_* is 100% (i.e., the electrical loss is zero), it is possible to estimate the effect of the VNHA structure and to compare the measured efficiency with optical modeling results. Therefore, PhOLEDs with an EQE of 29.1%, which were used as reference devices in this study, provide a good platform to validate the extent to which the VNHA structure contributes to the extraction enhancement due to their 100% internal efficiency. In addition, it is verified by comparing experimental and optical modeling results that the VNHA extracts the entire waveguide loss into the air.

## VNHA Fabrication

The VNHA substrate was fabricated using a novel process called robust reverse-transfer (R^2^T)^18^. An 800-nm-thick Si_3_N_4_ film was deposited onto a silicon substrate using plasma-enhanced chemical vapor deposition; then, a hexagonal hole array was introduced onto a silicon nitride film using conventional photolithography and dry etching. After the nanoholes were embedded, Si_3_N_4_ on the silicon substrate and the glass wafer were bonded in a vacuum using the anodic bonding procedure; the bonded wafers were then dipped in a KOH solution to remove the silicon substrate. A nanostructure slab was obtained and used as an OLED substrate with alternating vacuum holes (i.e., the low-index material) and Si_3_N_4_ matrices (i.e., the high-index material). Details of the VNHA structure fabrication method are given in a previous study^18^.

The R^2^T process was developed to maintain the nanohole array in the vacuum state. In general, because a top-to-bottom process fills a hole with the material from an upper layer, it is impossible to obtain an empty state. The periodic nanohole array is inserted in a vacuum state to maximize the refractive index (RI) contrast of the photonic crystal (PC) slab for the given high-RI background material. Thus, we transferred the periodic nanoholes onto a glass substrate while in a vacuum state. In addition, the substrate obtained from the R^2^T process has a smooth surface with a roughness of a few nanometers, which is comparable to that of a polished silicon wafer (see Fig. S1)^18^. Various methods were used to fabricate even surfaces for the inserted nanostructure, including plasma-enhanced chemical vapor deposition (PECVD)^19^,^20^, sol-gel spin-coating^21^,^22^, and the doctor blade process^23^. However, those surfaces were found to be wavy and rough beneath the inserted structure. A wavy device can enhance the efficiency by extracting both the waveguide and surface plasmon loss through Bragg reflection and localized surface plasmon resonance (LSPR) effects. However, a surface that is both wavy and rough can reduce the overall efficiency in an electrically optimized device because a well-matched charge balance on a flat device is easily broken by surface conditions^11^,^24^,^25^,^26^,^27^. Because the rough surface necessarily changes both the electrical and optical characteristics, it is not easy to identify the cause of the enhancement. However, optical analysis results are suitable for determining the performance of the VNHA-embedded device because the VNHA surface obtained by the R^2^T process affects only the optical characteristics. The surface roughness has a strong influence on the electrical characteristics of the device and is directly related to the device efficiency, which will be discussed in detail in the following sections.

## PhOLED Fabrication

**Figure 1:**
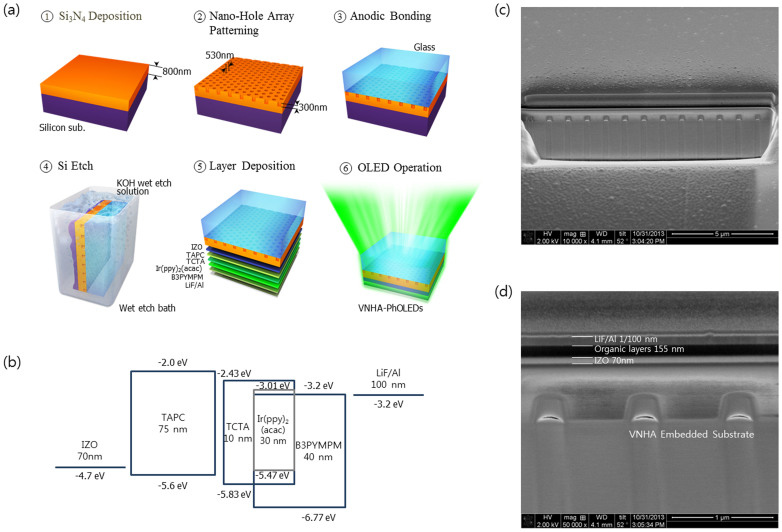
(a) Schematic diagram of the fabrication procedure of the vacuum nanohole array; (b) HOMO-LUMO energy level of the exciplex co-host phosphorescent OLEDs^3^; (c) and (d) Focused ion beam (FIB) images of the VNHA OLEDs fabricated by the R^2^T process and vertical structure of the VNHA-PhOLEDs.

OLEDs were fabricated on the VNHA and bare glass substrates. Figure 1 shows a schematic diagram of the R^2^T process, the device structure, and the molecular energy levels. A vacuum nanohole array substrate with a smooth surface was produced with the R^2^T process. For high efficiency, an exciplex-forming co-host system was formed in an excited state using 4,4′,4″-tris(N-carbazolyl)-tri-phenylamine (TCTA) and bis-4,6-(3,5-di-3-pyridylphenyl)-2-methylpyrimidine (B3PYMPM). This exciplex-forming co-host system enables efficient singlet and triplet energy transfers from the host exciplex to the phosphorescent dopant because the singlet and triplet energies of the exciplex are nearly identical^3^. Park et al. demonstrated that green phosphorescent OLEDs with high efficiencies using the exciplex of the co-host system have the aforementioned structure. The resultant device has a simple structure consisting of three transport organic materials and one phosphorescent-emitting dopant. The TCTA-B3PYMPM exciplex is characterized by a highly efficient transport charge, which is recombined in the confining zone of the emitting dopant, Ir(ppy)_2_(acac). The electrical loss including charge balance and exciton-polaron quenching of the device is negligible. Therefore, the device is useful in analyzing the effect of the inserted photonic structure on the extraction enhancement.

## VNHA PhOLEDs

As previously mentioned, the electrical loss should be zero if the experimental results are to be compared with those from optical modeling. The perfect device has the following features: (i) the charge balance should be matched perfectly at the emission layer through the selection of an optimized device structure (i.e., with an appropriate arrangement of charge transport materials with suitable thicknesses); (ii) low roll-off efficiency induced by 100% recombination over a large operation current range; and (iii) an extremely smooth surface for lossless and stable operation of the device. The PhOLEDs fabricated as a reference in this study already have a high efficiency of 29.1% EQE, and their low roll-off indicates a perfect electron and hole balance in the EML layer over the operation conditions considered. We obtained a VNHA-embedded substrate by transferring the VNHA, the top surface of which is extremely smooth, to the extent of the surface roughness that is comparable to that of a polished silicon wafer. Therefore, we can assume that the electrical loss of the VNHA PhOLEDs is negligible, which indicates that the experimental results can be compared with those from optical modeling.

## Results

### Mode Analysis

**Figure 2:**
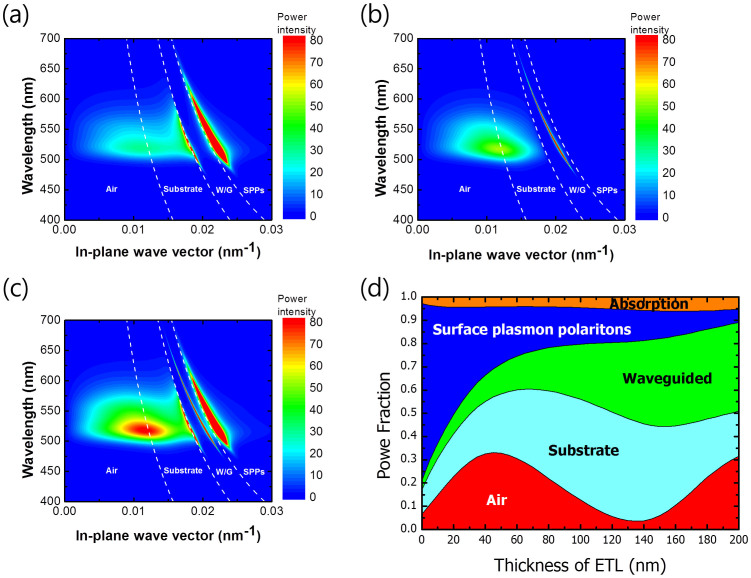
Mode simulation results of exciplex-forming co-host phosphorescent OLEDs with the power coupling ratio predicted using the classical dipole model for bottom emission: (a) P-polarized intensity (TM mode); (b) s-polarized intensity (TE mode); (c) total intensity with respect to the in-plane wave vector; and (d) power fraction conducted by changing the thickness of the electron transporting layer (EML).

Figure 2 shows the results of mode analysis based on the classical dipole model^28^,^29^. The non-isotropic dipole orientation factor of the emitting dye, Ir(ppy)_2_(acac), was applied to the simulation assuming that the charge balance was unity^30^. The mode analysis was conducted by changing the thickness of the EML, which is the most important cavity structure element with regard to layer thickness determination. Details of the simulation method have been previously given in the literature^31^,^32^. According to the results of the mode analysis, the EQE can be maximized with an emitting layer (ETL) of 40 nm thickness, where 32.2% is air mode extraction, 23.5% is lost through the substrate mode, 12.5% is lost through the waveguide mode, and 24.0% is lost through the surface plasmon mode. However, the light lost through the waveguide and substrate modes can be extracted to the air by inserting the VNHA structure. The contribution of each mode to the improvement in efficiency can then be identified by comparing the empirical and theoretical results due to the 100% internal efficiency of PhOLEDs with an EQE of 29.1%.

### Photoluminescence Performance

**Figure 3:**
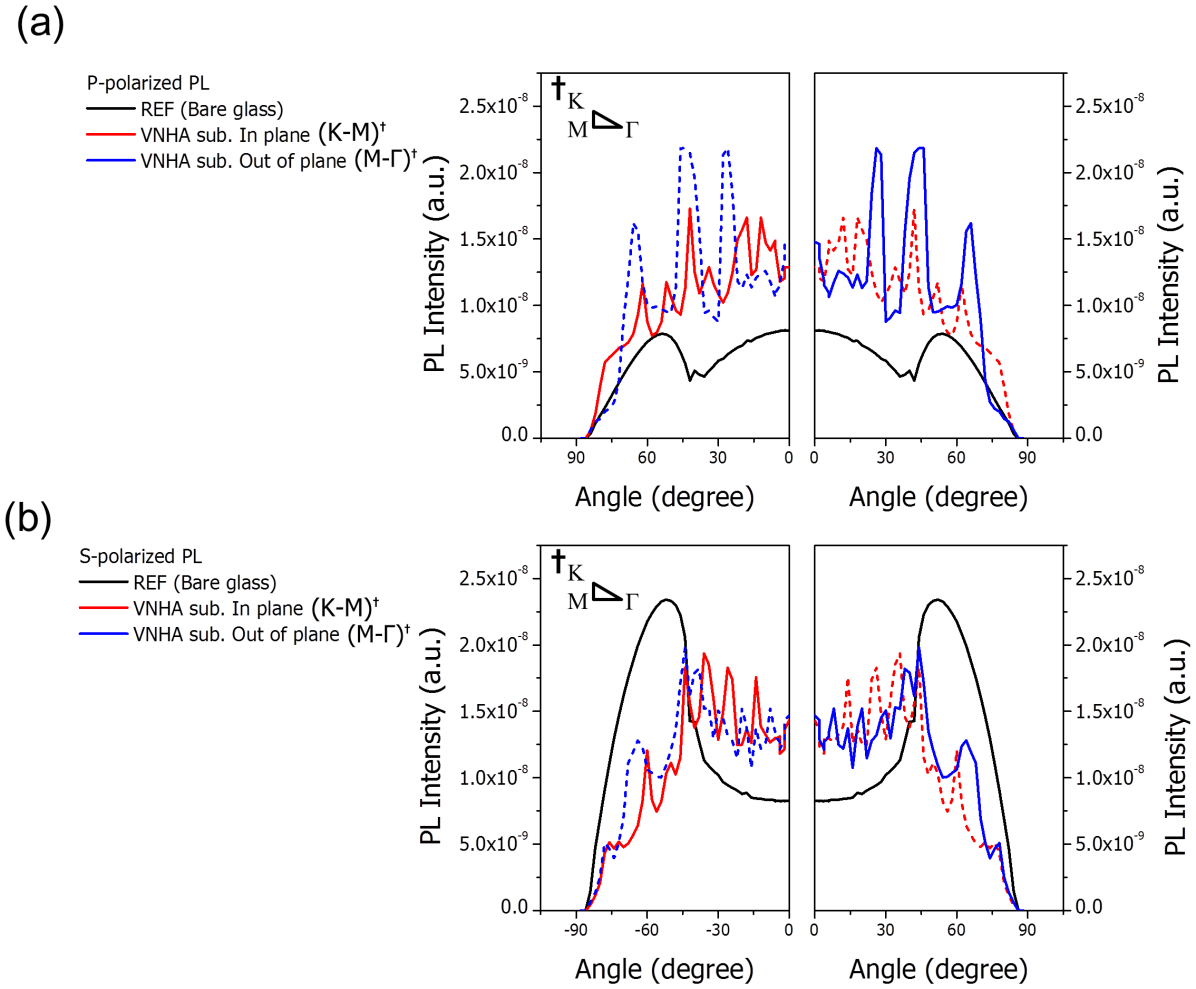
PL intensity measured as a function of the viewing angle for (a) p-polarized; and (b) s-polarized light emissions with a wavelength of 520 nm. The dotted lines represent the mirror image of the solid lines.

To confirm the optical enhancement of the vacuum nanohole array, the angular dependence of the photoluminescence (PL) is measured, as shown in Figure 3. The influence of the embedded structure on the horizontal (*dx*, *dy*) and vertical (*dz*) dipoles can be identified by observing the polarized angular PL. The in-plane and out-of-plane PLs are measured in 30-degree increments, and both should exhibit an increase if the enhancement effect of the inserted nanostructure is to be verified. If enhancement is observed in one direction only, it is likely that the intensity enhancement in that direction does not indicate an increase of light extraction to the air but rather a change in the light path.

Figure 3 shows the p- and s-polarized PL intensities depending on the viewing angle. The *dy*-dipoles emit only s-polarized light, whereas the *dx*- and *dz*-dipoles are responsible for the p-polarized emission^33^. Hence, the angle-dependent PL spectrum was measured to determine the exact fraction of horizontal and vertical dipoles based on that of an isotropic molecule. The phosphorescent emitter, Ir(ppy)_2_(acac), has a horizontally preferred non-isotropic dipole orientation with a horizontal-to-vertical dipole ratio of 0.77:0.23, whereas the isotropic random orientation has a ratio of 0.67:0.33^30^. The PL intensity measurement indicates that enhancement is induced only by the waveguide mode resulting from the spherical interface between glass and air. The PL measurement only considers the optical effects of the VNHA structure because such electrical effects as charge balance and surface plasmon polaritons cannot be involved. As shown in Figure 3, the PL intensities of the s- and p-polarized light increased significantly at the critical angle range of ± 41.8° for the interface between glass (n = 1.47) and air (n = 1.0). Therefore, the proposed VNHA structure provides optical enhancement with regard to emission extraction induced by both vertical and horizontal dipoles.

On the other hand, the p- and s-polarized PLs of the fluorescent isotropic dipole emitter, Alq_3_, were also measured in a previous report^18^. Although those two emitters, Alq_3_ and Ir(ppy)_2_(acac), differ in terms of molecular structure, PL efficiency and emission type (i.e., fluorescent and phosphorescent, respectively), the measurement results show that their angular PL peaks appear at the same viewing angle. This indicates that the optical characteristics of the given photonic structure are determined by the wavelength of the emitted light; however, the enhancement levels are slightly different. Although the band gaps of Alq_3_ and Ir(ppy)_2_(acac) are identical and their PL spectra are similar, Ir(ppy)_2_(acac) has a horizontally preferred dipole and the p-polarized intensity enhancement responsible for *dz* decreases as the number of horizontal dipoles increases.

### Electroluminescence Performance

**Figure 4:**
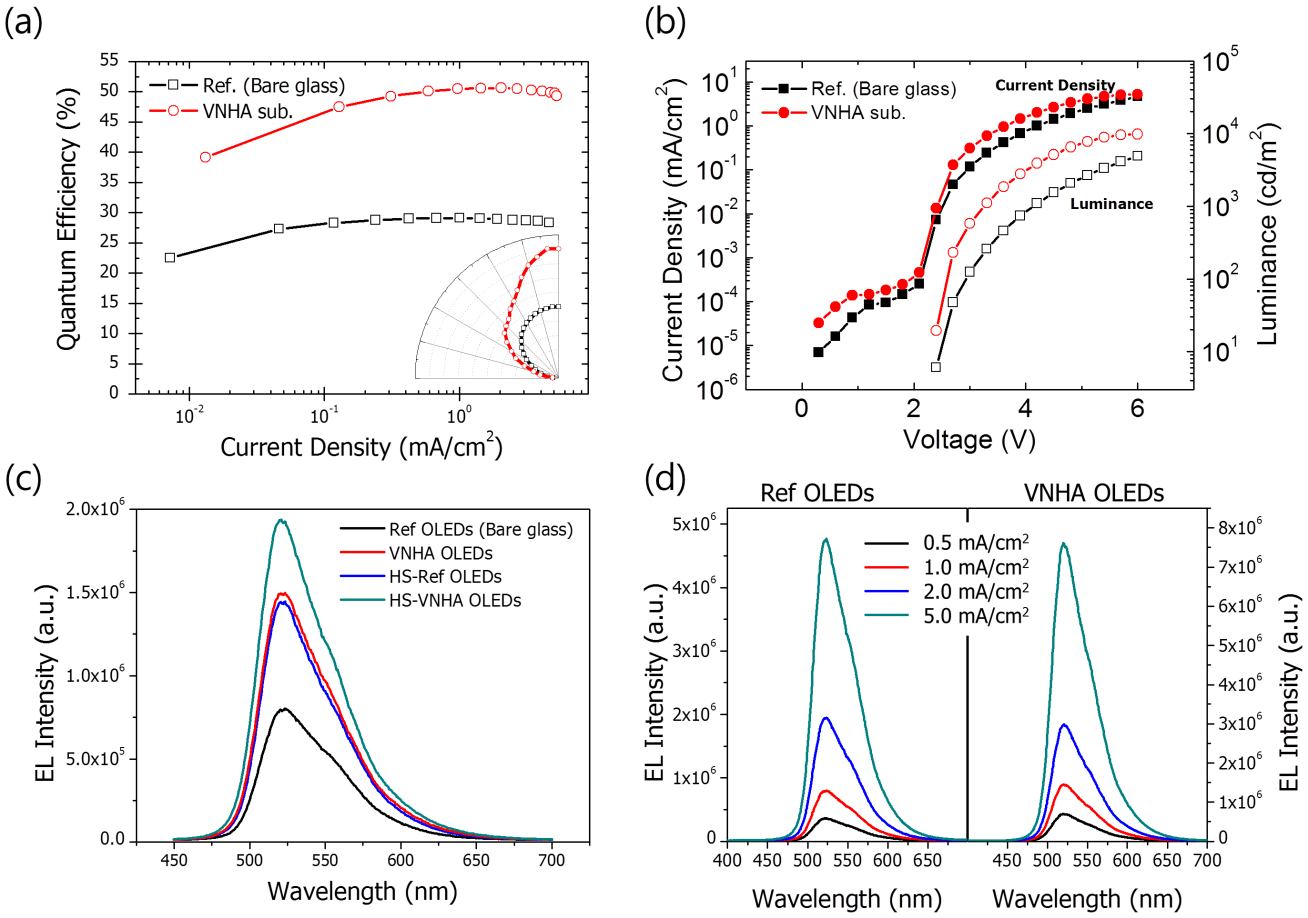
(a) External quantum efficiencies (inset: angular dependent emission); (b) power efficiencies of the reference and VNHA PhOLEDs; (c) EL intensity measured for each device as a function of wavelength. The EL intensities of the VNHA-PhOLEDs (red line) and the control (black line) devices were measured using an integrating sphere. The EL intensity was also measured for each device by attaching a hemisphere lens with a 10 mm diameter to the VNHA-PhOLED (blue line) and control (green line); (d) EL intensity under the different current densities of 0.5, 1.0, 2.0 and 5.0 mA/cm^2^.

Figure 4 shows the EL performance of the VNHA-embedded OLEDs. The phosphorescent OLED used as a reference has an ultrahigh efficiency of 29.1% that corresponds to an internal quantum efficiency (IQE) of 100%. This efficiency is in good agreement with the mode analysis results calculated without considering electrical factors. The low roll-off indicates that the charge balance factor must be near unity throughout a broad range of current density and that electrical loss is negligible. The reference OLED is ideal as a platform for an analysis of the extraction factor of the VNHA structure and is also useful for understanding the mode analysis results. The VNHA obtained from the R^2^T process provides a smooth surface with the lowest surface roughness, as mentioned previously, and thereby leads to a negligible electrical loss. The EL enhancement was measured by an intensified CCD, as shown in Figures 4(c) and 4(d). The relative spectra were measured using an integrating sphere to detect the light radiated in all directions under the same current (e.g., 1.0 mA/cm^2^) at which the EQE of the reference device reached a maximum. The relative spectrum of the VNHA embedded green PhOLEDs was found to be improved by 1.74 times, and the resultant EQE was 50.7%. This EQE is slightly higher than that of the reference OLEDs with hemisphere lenses attached to the top surface (HS-OLEDs). The VNHA OLEDs are brighter than the HS-OLEDs that achieved a maximum EQE value by extracting all trapped light in the glass substrate. The EQE of the VNHA OLEDs was obtained through calibration using the relative spectra measured under varying current densities, as shown in Figure 4(d). The spectrum did not change with increasing current density, as shown in the figure; this indicates that the emission zone did not vary. The radiation pattern given in the inset of Figure 4(a) was measured at 1 mA/cm^2^, and the radiation may retain the same pattern with increasing current density.

In addition, the reference and VNHA devices showed the same turn-on voltage of 2.4 V, as shown in Figure 4(b). This indicates that both devices have the same injection conditions, but the slightly higher current density of the VNHA device causes the surface condition. The VNHA substrate obtained by the proposed R^2^T process has a smooth surface that is comparable to the surface roughness of polished Si wafers; for comparison, the surface roughness (R_a_) of bare glass (BOROFLOAT®33) is less than 3 nm, and that of the VNHA substrate is approximately 3 Å. Surface conditions can affect the contact resistance of the layer interface, which may affect the series resistance of the device. The VNHA substrate influences the optical characteristics and electrical performance slightly. The VNHA substrate is useful in practice because the power efficiency remains high at higher current densities.

The extraction of the waveguide mode can also be measured by attaching a hemisphere lens, which can avoid total internal reflection at the glass-air interface. Thus, a hemisphere lens with a diameter of 10 mm is attached to the glass surface with index matching oil. The spectra of each device with a hemisphere lens attached to its surface can then be measured to identify the enhancement caused by the waveguide mode alone because the glass-air interface loss is negligible as a result of the presence of the hemisphere lens. The enhancement factor values and corresponding EQEs are summarized in Table 1. As indicated in the table, the HS-VNHA OLEDs exhibit an approximately 17.17% higher EQE compared with the reference HS-OLEDs, which indicates that the EQE of the VNHA OLED is increased by the amount equivalent to the sum of both the light loss due to the waveguide mode and the partial loss of the surface plasmon and substrate modes. Although the surface plasmon mode is not controlled directly, the VNHA structure can contribute to the reduction of the surface plasmon loss by extracting the light emitted from the vertical dipoles. Nearly all of the light emitted from the vertical dipoles is dissipated at the metal surface, but the light from the vertical dipoles is substantially extracted in the case of the VNHA device. This is confirmed by the results of the mode analysis, angular PL, and FDTD simulations.

To numerically analyze the device embedded in two dimensional arrays, we conducted an FDTD simulation with respect to the refractive index contrast in a previous report^18^. (See Fig. S5) As shown in Figure 2(a), surface plasmon loss is observed only in the p-polarized light induced from d_x_ and d_z_, and the p-polarized light can be significantly enhanced by the VNHA structure, as demonstrated by the angular PL (Figure 3(a)). The FDTD simulation showed that the VNHA structure effectively extracted light from the vertical dipoles. Once the light escapes from the organic layer, surface plasmon loss can be avoided. The loss induced by the surface plasmon mode in the bare substrate is reduced because the light escaping the organic layer through the high index layer and the VNHA structure results in a decrease in the amount of light in contact with the metal surface at the bottom. A comparison of the FDTD, mode analysis, and angular PL results illustrates that the enhanced EQE due to the VNHA structure is primarily caused by the extraction of the waveguide mode and is partially attributed to the extraction of the light emitted from the vertical dipoles.

## Conclusion

We obtained the highest-efficiency OLED that yields both an EQE of over 50% and a low roll-off by inserting a VNHA into PhOLEDs. The R^2^T process was utilized to generate the nanohole array, which was then inserted to the PhOLED in a vacuum to maximize the refractive index contrast of the PC slab for a given background material of high RI. The EQE obtained in this study is the highest value obtained for bottom-emitting OLEDs to date. The measured performance of the VNHA OLEDs was compared with optical modeling analysis results, and the VNHA was verified to extracts the entire waveguide loss into the air.

## Method

### OLED device fabrication

An indium zinc oxide (IZO) anode with a thickness of 70 nm^31^ was sputtered on the substrate, and the organic and metallic layers were deposited sequentially by thermal evaporation as follows: a 75 nm-thick hole injection layer of 1,1-bis-(4-bis(4-methyl-phenyl)-amino-phenyl)-cyclohexane (TAPC), a 10 nm-thick hole transporting layer of 4,4′,4″-tris(N-carbazolyl)tri-phenylamine (TCTA), an emitting layer of bis(2-phenylpyridine)iridium(III)-acetylacetonate (Ir(ppy)_2_(acac))-doped TCTA:B3PYMPM [bis-4,6-(3,5-di-3-pyridylphenyl)-2-methylphyrimidine], a 40 nm-thick electron transport layer of B3PYMPM, and a 1/100 nm-thick cathode of LiF/Al. Prior to organic layer deposition, the IZO substrates were exposed to UV-ozone flux for 10 min. The layers were then deposited by thermal evaporation at a base pressure of less than 5 × 10^−7^ Torr while still under vacuum.

### Angular-dependent PL measurements

The angular-dependent PL was measured using a continuous wave diode laser (325 nm, Edmund Optics Inc.). The incident angle of the excitation source was fixed at 45°, and s- and p-polarized emitted light was detected at 520 nm, which is near the peak wavelength of the PL spectra of the phosphorescent dopant. A schematic diagram of angular PL intensity measurements is given in supplementary information. (See Fig.S6)

### EL measurements

The current density, voltage, and EL luminance were measured using a Keithley 2400 programmable source meter and SpectraScan PR650 (Photo Research). The angular distribution of the EL intensity was measured using the Keithley 2400 programmable source meter, a rotation stage, and an Ocean Optics S2000 fiber optic spectrometer.

The EL intensities were measured using an integrating sphere (Labsphere Co., 6-inch diameter) and a Keithley 2400 source meter. To detect all light emitted from the devices in the forward direction, the OLEDs were attached to a port of the integrating sphere with the glass side facing the interior of the sphere. An intensified charge-coupled device (ICCD) was used as the optical detector for the measurement, and the spectrum was detected and integrated by the ICCD 100 times.

The EQE of the control device fabricated on the bare glass was calculated from the current density, luminance, EL spectra, and angular distribution of the EL intensity data. It is difficult to obtain an accurate three-dimensional radiation profile for VNHA OLEDs. For optimal accuracy, the EQE was calculated from the measured relative spectra of the reference OLEDS and VNHA OLEDs, measured using an ICCD and the integrating sphere at the same current density of 1 mA/cm^2^.

## Author Contributions

The manuscript was written through contributions of all authors. S.J. performed all experiments and the numerical simulations, analyzed the data, designed the system and wrote the paper. J.H.L. fabricated and measured the electroluminescence and photoluminescence devices, and C.K.M. performed the mode analysis. J.J.K. designed the device structure, provided the experimental set-up and discussed the results. Y.S.S. analyzed the data and discussed the results. J.H.J. and J.R.Y. led the overall direction of the project. All authors have given their approval to the final version of the manuscript.

## Supplementary Material

Supplementary Information Supplementary information

## Figures and Tables

**Table 1:** EL enhancement values and EQEs for different wavelength ranges

	At 520 nm	505–565 nm	280–700 nm	EQE
	Max. peak	FWHM	Visible Range	(%)
Reference	1.0	1.0	1.0	29.11
VNHA-OLEDs	1.86	1.80	1.74	50.65
HS-Reference	1.81	1.74	1.72	50.07
HS-VNHA-OLEDs	2.45	2.35	2.31	67.24
