# Supplementary material for: Vacuum Nanohole Array Embedded Phosphorescent Organic Light Emitting Diodes
Source: Sci Rep. 2015 Mar 3;5:8685. doi: 10.1038/srep08685 (PMC4346830; doi:10.1038/srep08685)
Supplement: Supplementary Information [file srep08685-s1.doc]

**Supplementary Information**

Vacuum Nanohole Array Embedded Phosphorescent Organic Light Emitting Diodes

Sohee Jeon, ^1, 2^ Jeong-Hwan Lee, ^1^ Jun-Ho Jeong, ^2^ Young Seok Song, ^3^ Chang-Ki Moon, ^1^ Jang-Joo Kim, ^1^ and Jae Ryoun Youn^*1^

^1^Research Institute of Advanced Materials (RIAM), Department of Materials Science and Engineering, Seoul National University, Seoul, 151-744, Korea
^2^Department of Nano Manufacturing Technology, Nano-Convergence Mechanical Systems Research Division, Korea Institute of Machinery and Materials, Daejeon, 305-343, Korea
^3^Department of Fiber System Engineering, Dankook University, Yongin, Gyeonggi, 448-701, Korea

* Correspondence and requests for materials should be addressed to Jae R. Youn ([jaeryoun@snu.ac.kr](mailto:jaeryoun@snu.ac.kr)).


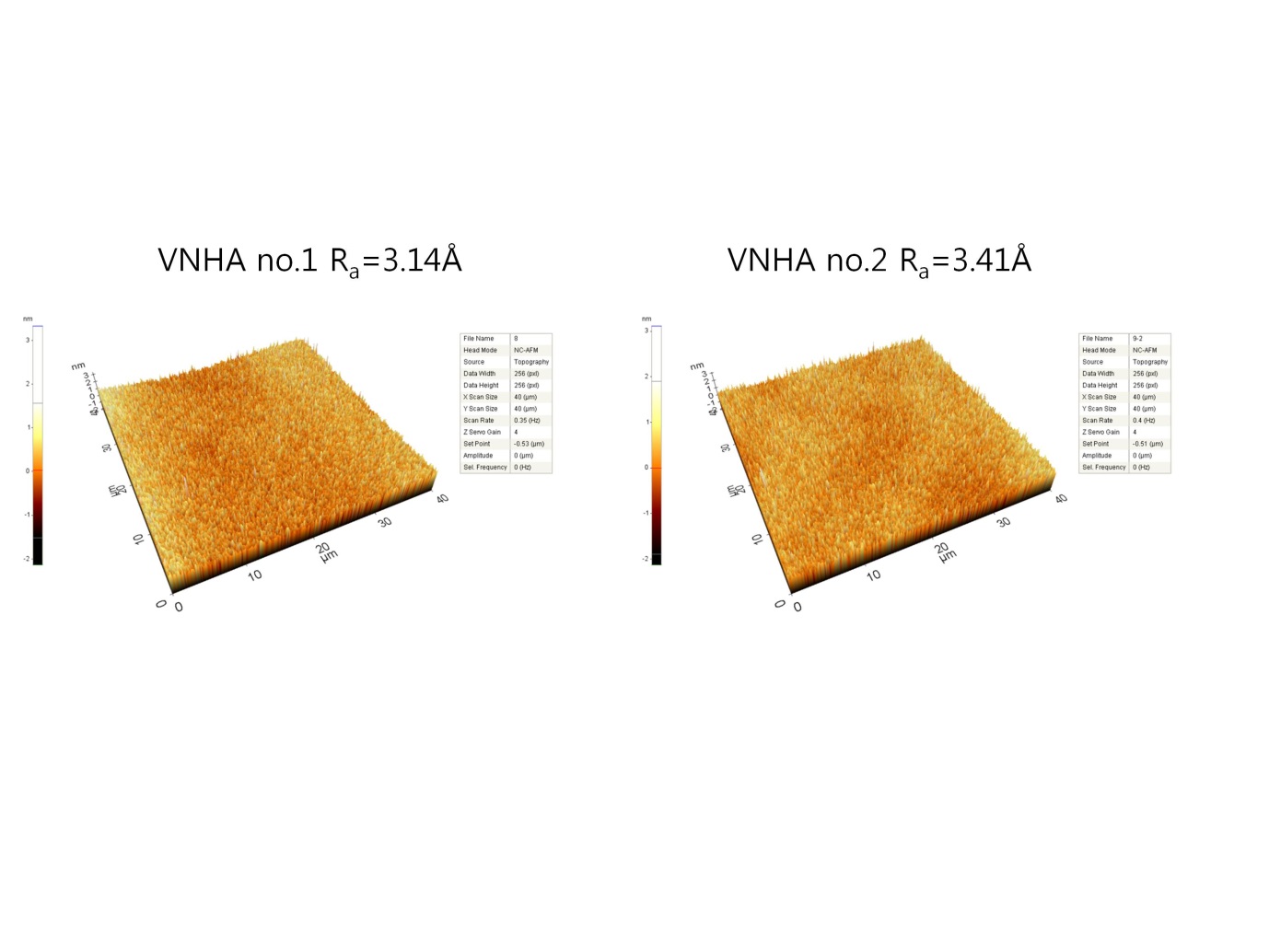
Figure S1. Atomic force microscopy (AFM) images of the surface of VNHA substrate


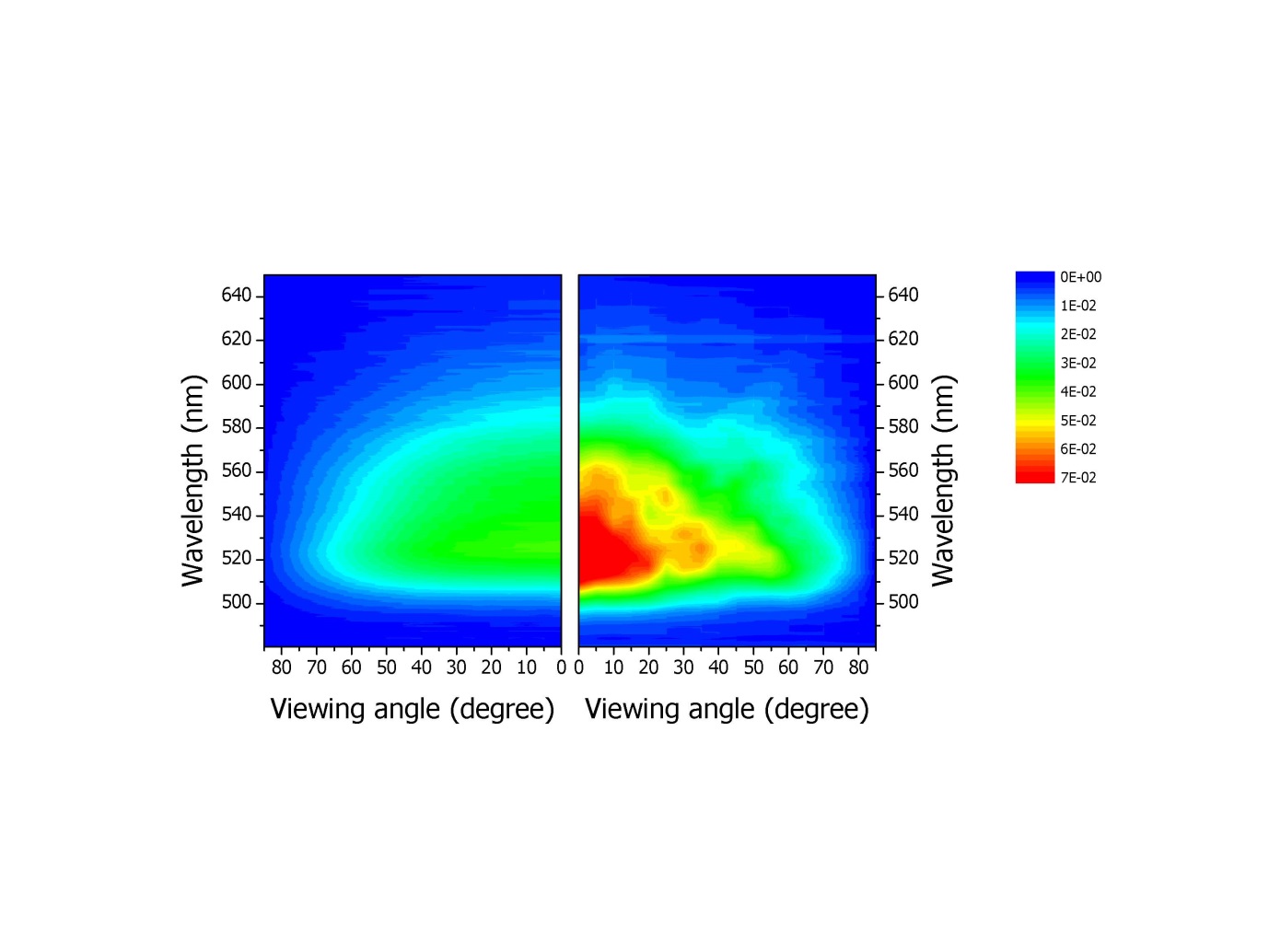
Figure S2. Full EL intensity contours of the reference OLEDs (left) and VNHA-OLEDs (right) depending upon the wavelength and the viewing angle


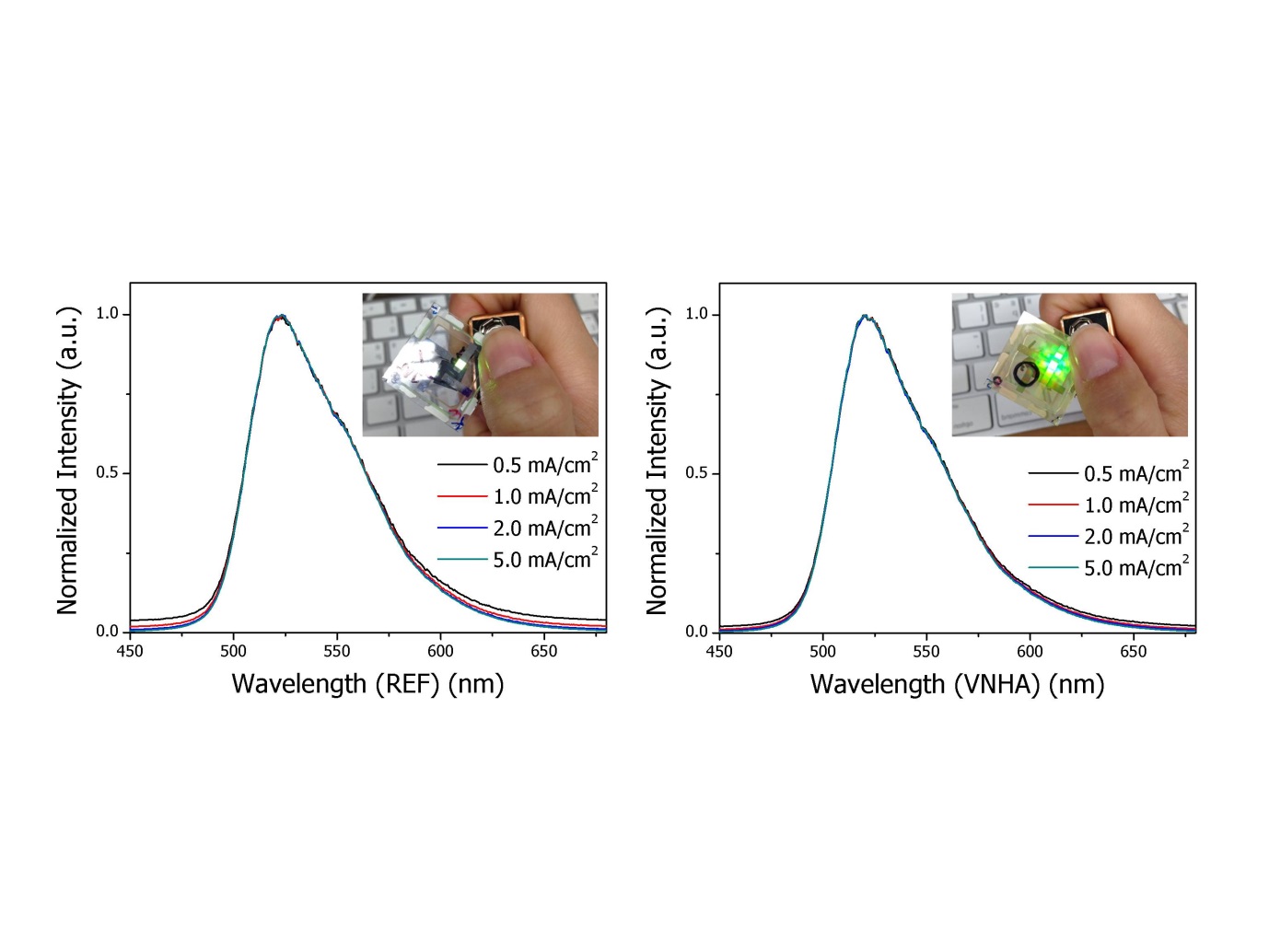
Figure S3. Normalized Intensities and photographs of reference OLEDs (left) and VNHA OLEDs (right)


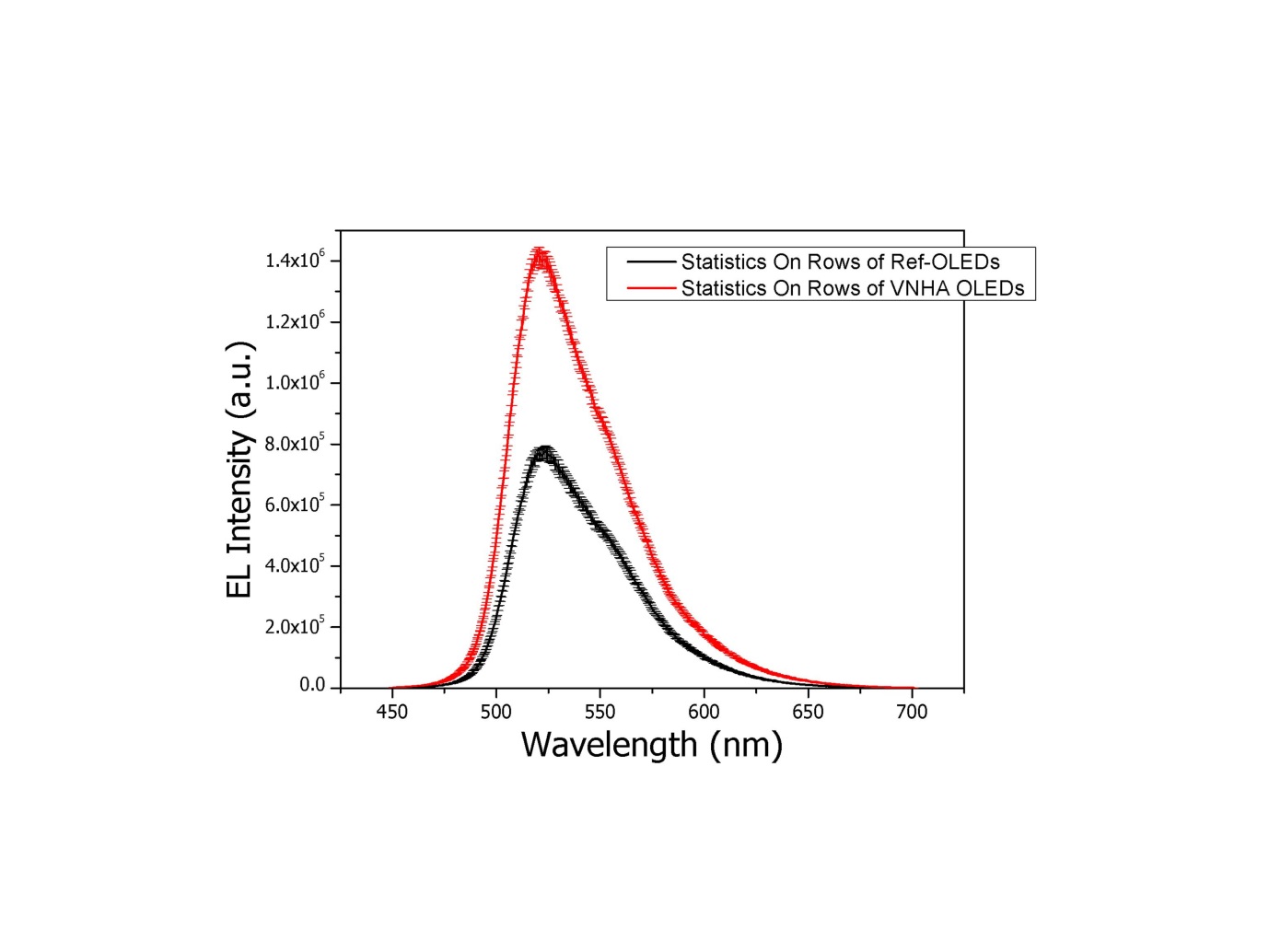
Figure S4. The standard deviation of the control group EQE is 2.0% and that of the VNHA OLEDs is 1.4%.


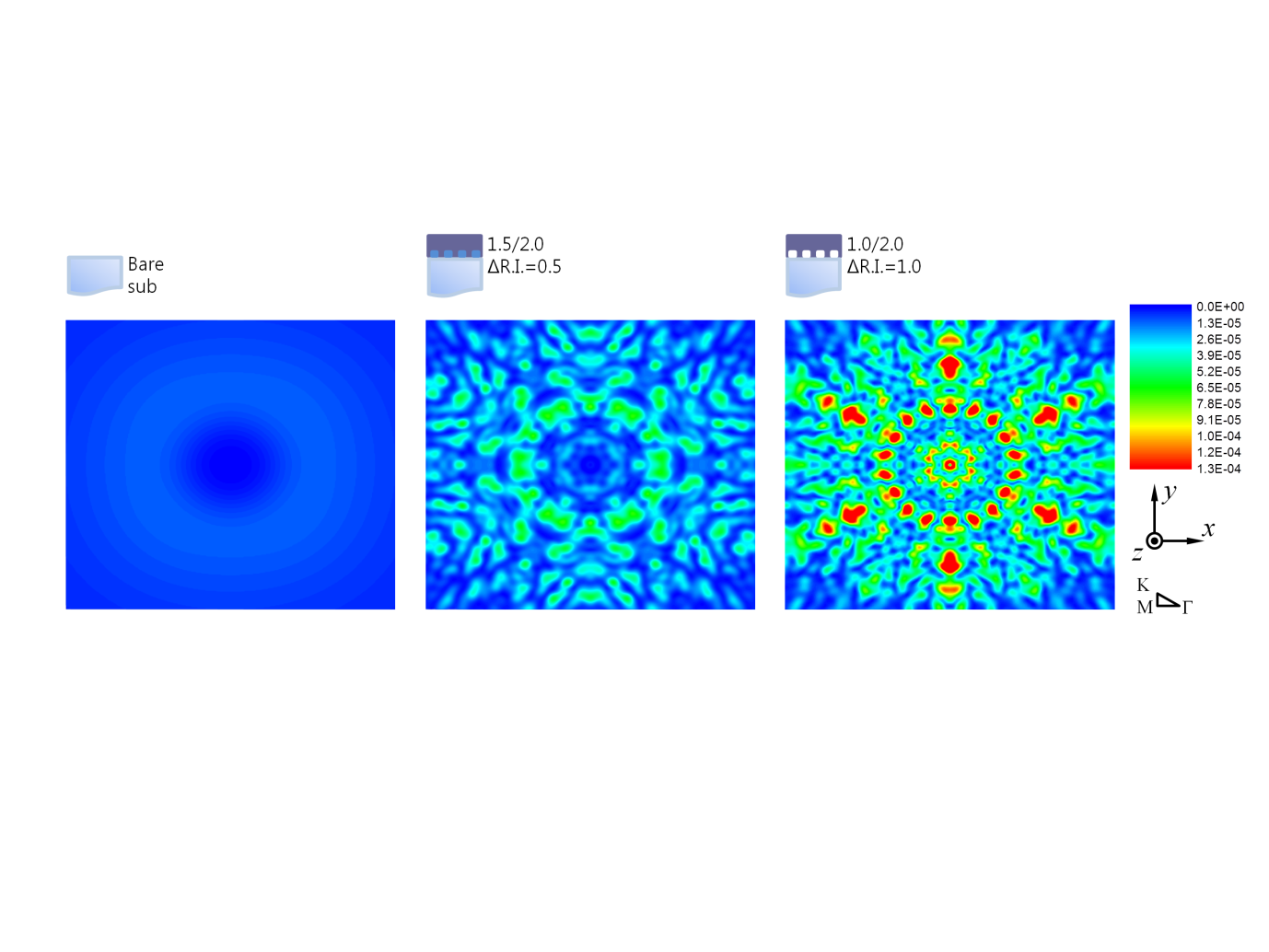


Figure S5. FDTD analysis results: contours of Poynting energy emitted from vertical dipole on the bare glass (reference), 1.5/2.0 nanohole array, and 1.0/2.0 nanohole array substrates


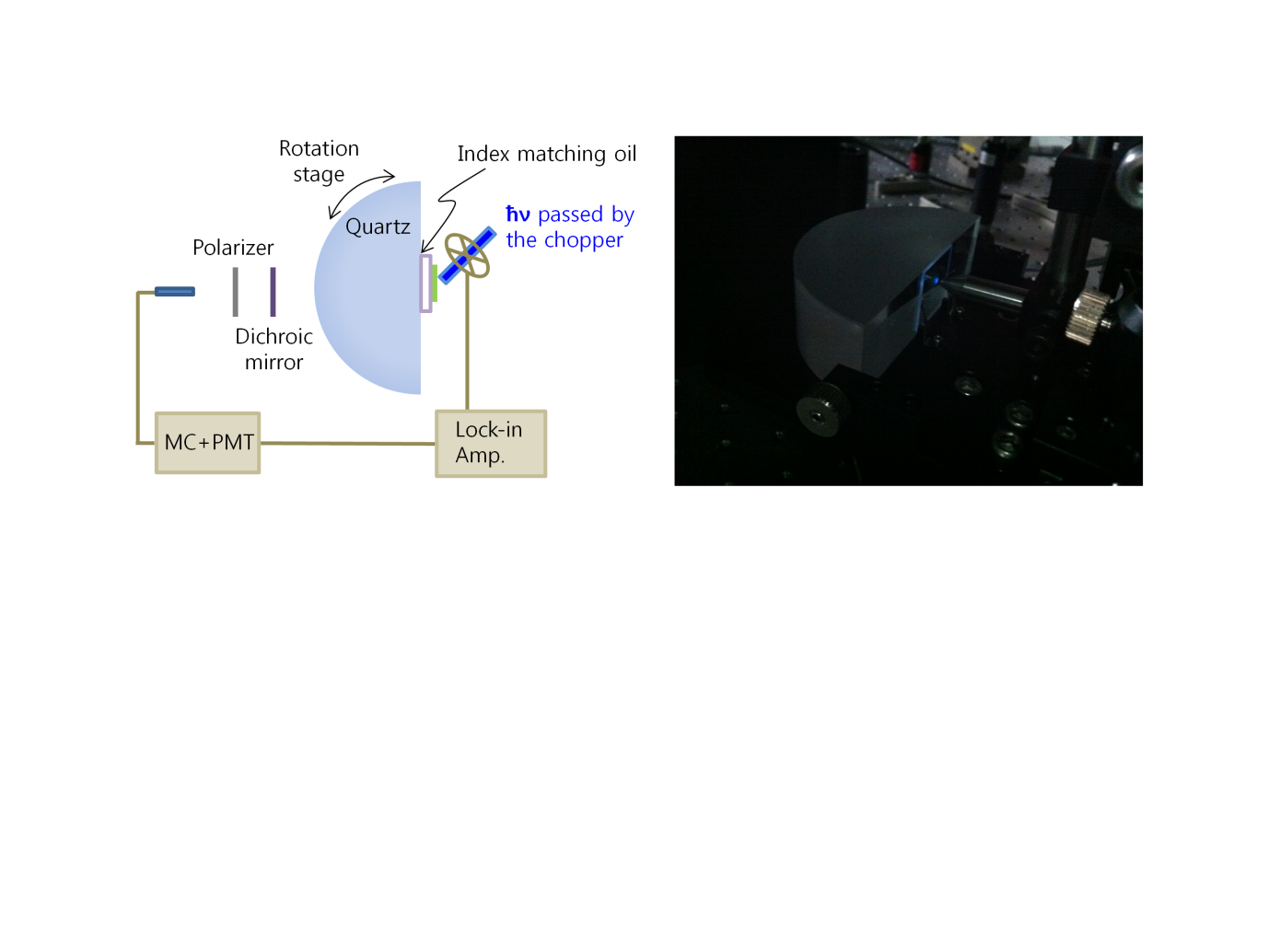


Figure S6. Schematic diagram and photograph of the angular dependent PL measurement set-up
